# Immune Cell Infiltration of the Primary Tumor, Not PD-L1 Status, Is Associated With Improved Response to Checkpoint Inhibition in Metastatic Melanoma

**DOI:** 10.3389/fmed.2019.00027

**Published:** 2019-03-13

**Authors:** Christiane Kümpers, Mladen Jokic, Ozan Haase, Anne Offermann, Wenzel Vogel, Victoria Grätz, Ewan A. Langan, Sven Perner, Patrick Terheyden

**Affiliations:** ^1^Pathology of the University Hospital Schleswig-Holstein, Luebeck and Research Center Borstel, Leibniz Lung Center, Luebeck, Germany; ^2^Department of Dermatology, University of Luebeck, Luebeck, Germany; ^3^Department of Dermatological Sciences, University of Manchester, Manchester, United Kingdom

**Keywords:** melanoma, PD-L1, immunoscore, checkpoint inhibition, lymphocyte, metastases, checkpoint inhibitor therapy

## Abstract

Immune checkpoint inhibition has resulted in dramatic improvements in overall and relapse-free survival in patients with metastatic melanoma. The most commonly used immune checkpoint inhibitors are monoclonal antibodies targeting programmed cell death protein 1 and cytotoxic T-lymphocyte-associated protein 4. Unfortunately, a significant subset of patients fail to respond to these therapies, which has resulted in intense research efforts to identify the factors which are associated with treatment response. To this end, we investigated immune cell infiltration in primary melanomas and melanoma metastases, in addition to tumor cell PD-L1 expression, to determine whether these factors are associated with an improved outcome after immune checkpoint inhibition. Indeed, the extent of the immune cell infiltration in the primary melanoma, measured by the Immunoscore, was associated with a significantly improved response to immune checkpoint inhibition in terms of increased overall survival. However, the Immunoscore did not predict which patients would respond to treatment. The Immunoscore was significantly reduced in metastases when compared to primary melanomas. In contrast, PD-L1 expression, exhaustively tested using four commercially available anti-PD-L1 clones, did not differ significantly between primary tumors and melanoma metastases and was not associated treatment response. Whilst replication in larger, prospective studies is required, our data demonstrates the relevance of immune cell infiltration in the primary melanoma as a novel marker of improved overall survival in response to immune checkpoint inhibition.

## Introduction

Although melanoma is highly refractory to treatment with conventional chemotherapy, the advent of immune checkpoint inhibition has dramatically improved the clinical outcome in metastatic disease (1). Immune checkpoint inhibition in melanoma relies on the use of antibodies blocking either the programmed cell death protein 1 (PD-1), for example Nivolumab and Pembrolizumab, preventing melanoma tumor cells from escaping toxic T-cell action, or antibodies targeting the cytotoxic T-lymphocyte-associated protein 4 (CTLA-4), namely Ipilimumab, leading to prolonged T-cell activation and resulting in clonal expansion and enlarged T-cell repertoire. Whilst immune checkpoint inhibition has been associated with impressive long-term response rates, there remains a subset of patients who either fail to respond to therapy (primary resistance), or lose the initial response (secondary resistance) during treatment (2).

Therefore, current research efforts are focused on identifying factors associated with treatment response in order to individually tailor treatment (3). For example, an increased tumor mutational load is associated with improved outcome under checkpoint inhibition, potentially via the induction of immune cells which differentially recognize tumor- from normal cells (4, 5). On the other hand, melanoma can express a specific mutational profile which is able to induce an innate anti-PD1 resistance (IPRES) phenotype, rendering the melanoma effectively unresponsive to immune checkpoint inhibition (6).

The expression of programmed cell death ligand 1 (PD-L1) in melanoma is perhaps the most intensively studied marker of response to treatment with checkpoint inhibition (7, 8). In a comprehensive review of biomarkers for response of melanoma to checkpoint inhibition, Jessurun et al. found a significant correlation between tumor PD-1 and PD-L1 expression and response to checkpoint inhibition in five out of eight analyses. Interestingly, there was no significant correlation with progression-free survival. Whilst divergent methodology may make comparison of these studies difficult, it is clear that overall response, progression-free survival and overall survival are not synonymous, and were correctly reported separately. Moreover, prognostic markers are not necessarily predictive markers of response to treatment (8).

Ultimately, whilst PD-L1 status has been shown to correlate with response to treatment with anti-PD-1 antibodies in metastatic melanoma in some studies (9, 10), the expression of PD-L1 *per se* has not emerged as a predictive marker for treatment response, potentially due to its crucial role in engaging PD-1, a dominant negative regulator of anti-tumor T cell effector function (1, 9, 11). In the clinical setting, PD-L1 expression cannot be relied upon as a predictive marker of treatment response, given that not all tumors expressing PD-L1 respond to PD- inhibitors (12) and melanomas with little or no PD-L1 expression may still respond to checkpoint inhibition.

In contrast, pre-existing tumor immune cell infiltration is considered to be an important factor determining successful immune checkpoint inhibition and consequently treatment response (13). Melanoma is recognized as a tumor that is often infiltrated with immune cells; the grade of tumor-infiltrating lymphocytes being an independent predictor of survival irrespective of the treatment type (14–17). Given the immunogenic nature of melanoma (18), as well as the poor prognosis associated with metastatic disease, we sought to objectively determine the immune cell infiltration (Immunoscore) and PD-L1 status of both primary tumors and metastases in a retrospective cohort based study of patients with metastatic melanoma, treated with anti-CTLA-4 and/or anti-PD-1 antibodies. The Immunoscore captures the number und distribution of tumor-infiltrating lymphocytes and was first described by Clark et al. (19) The grade of tumor-infiltrating lymphocytes is defined as either brisk, nonbrisk or absent. Given the range of commercially available anti-PD-L1 antibodies, we also investigated antibody specificity before utilizing the optimal antibody for the immunohistochemical staining. Finally, we addressed the question of whether immune cell infiltration and/or PD-L1 status of primary melanomas and metastases were associated with the clinical response, specifically in terms of overall survival, to immune checkpoint inhibition.

## Materials and Methods

### Study Population/Case Selection

The patient cohort comprised 32 patients (25 male, 7 female), who were diagnosed with metastatic melanoma and treated with checkpoint inhibitors at the Department of Dermatology, University of Luebeck. Patients underwent treatment with CTLA-4-inhibition (Ipilimumab) and/or anti-PD1-therapy (Nivolumab or (Pembrolizumab). 2 Patients were treated with Ipilimumab monotherapy. 12 patients were treated with Nivolumab (*n* = 6) or Pembrolizumab (*n* = 6). 11 patients received Ipilimumab prior to anti-PD-1-therapy, 4 patients received Ipilimumab prior to combined therapy with Ipilimumab and a PD-1-inhibitor and 3 patients initially received combination therapy with Ipilimumab and a PD1-inhibitor followed by a PD-1-inhibitor (Table 1).

**Table 1:** Patients' baseline characteristics.

**SEX**	
male	25
female	7
**AGE AT DIAGNOSIS (YEARS)**	
mean	64
range	32-91
**VITAL STATUS AT LAST FOLLOW UP**	
alive	9
dead	23
**IMMUNE CHECKPOINT INHIBITOR THERAPY**	
Ipilimumab mono	2
Nivolumab mono	6
Pembrolizumab mono	6
first Ipilimumab, afterwards PD-1-Inhibitor	11
first Ipilimumab, afterwards combinated therapy	4
first combinated therapy, afterwards PD-1-Inhibitor	3
**OVERALL SURVIVAL (DAYS)**	
mean	1272
range	31-3527
**PROGRESSION FREE SURVIVAL**	
mean	194
range	3-1310
**INTERVAL BETWEEN DIAGNOSE AND FIRST DOSE OF PD-1-INHIBITOR (DAYS)**	
mean	862
range	14-3425
**BRAF-MUTATION STATUS**	
wildtype	20
mutation	12
**COMPOSITION OF FFPE MATERIAL**	
cases with tissue from primary tumor and metastases	19
cases with tissue solely from primary tumors	3
cases with tissue solely from metastases	10
number of all metastases samples	88
number of naive metastases	54
number of metastasespost anti-PD1-therapy	20
number of metastases post Ipilimumab	14
**TIL GRADE IN PRIMARY TUMORS**	
non-brisk	9 (41%)
brisk	13 (59%)
**TIL GRADE IN PRIMARY METASTASES**	
non-brisk	37 (68,5%)
brisk	17 (31,5%)
**TIL GRADE IN RELAPSED METASTASES (AFTER ANTI-PD1-THERAPY)**	
non-brisk	16 (80%)
brisk	4 (20%)

The median age at time of diagnosis was 64 years. Nine patients remained alive at the last follow up point. Tissue blocks were retrieved from the archive, having been initially obtained between 2006 and 2016.

Out of the 32 patients, we retrieved primary tumor tissue from 22 patients, while from 10 patients only metastatic tissue was available. From a total of 22 patients for whom primary tumor samples were available, corresponding metastatic tissue was available from 19 cases. Out of the 19 patients with primary and metastatic lesions, 15 had metastatic lesions obtained prior to initiation of anti-PD-1-therapy (matched pairs). Up to 9 metastases (distant and/or lymph node) were available per patient.

Primary tumors, as well as lymph node and distant metastases, obtained before and after immune checkpoint inhibitor therapy were analyzed separately. The “tumor groups” were classified as follows (i) primary tumors (22 patients), (ii) distant metastases obtained pre-treatment (15 patients), (iii) lymph node metastases obtained pre-treatment (12 patients), (iv) distant metastases obtained during treatment (7 patients) and (v) lymph node metastases obtained during treatment (1 patient).

Baseline characteristics of the cohort including sex, age at diagnose, vital status at last follow up, treatment, overall survival, progression free survival, interval between diagnose and first dose checkpoint inhibitor, composition of FFPE material and the Immunoscore of primary tumors and metastases were recorded (Table 1). Observation time was the interval from the date of diagnosis to the date of last follow-up or death. Overall survival and progression-free survival ranged from 31 to 3,527 days (mean 1272 days) and from 3 to 1,310 days (mean 194 days), respectively.

Ethical approval for using human material in this study was obtained from the Internal Review Board of University of Luebeck (17–186). All data were anonymized before included to this retrospective study cohort.

### Histopathological Analysis

Formalin-fixed paraffin-embedded (FFPE) tissue blocks were retrieved from the archives of the Department of Pathology of the University Hospital Schleswig-Holstein, Campus Luebeck and Research Center Borstel, Leibniz Lung Center, Site Luebeck, the Clinic for Dermatology of the University Hospital Schleswig-Holstein, Campus Luebeck. Tissue microarrays (TMA) were constructed from metastatic samples in triplicates of 0.6 mm diameter cores. A tumor sample was included for further investigation if at least two cores were evaluable. Values of protein expression generated by Immunohistochemistry (IHC) for all examined cores of a patient sample were recorded as a mean value. The TMA included 74 samples of metastatic lesions from 24 patients. Tissue from 14 metastases (from 9 patients) was too small for TMA and therefore investigated as a whole section. All primary tumors were investigated as a whole section due to the small tumor size in most cases. Evaluation of protein expression by IHC was performed by two independent pathologists (CK, SP) who were blinded to the clinico-pathological data.

### Immunohistochemical Analysis

Immunohistochemical (IHC) staining was performed using the Ventana Discovery (Ventana Medical System) automated staining system. In brief, slides were incubated at room temperature with the following primary antibodies (dilution, clone, company): anti-PD-L1 (1: 50, E1L3N, Cell Signaling), anti-PD-L1 (RTU, SP263, Roche), anti-PD-L1 (RTU, SP 142, Roche), anti-PD-L1 (1:100, 28.8, Abcam), anti-PD-L2 (1:100, OTI6C3, Acris), anti-PD-1 (RTU, NAT105, Roche), anti-CD8 (RTU, SP57, Roche), anti-CD4 (RTU, SP35, Roche), anti-CD56 (RTU, MRQ-42, Roche), anti-FoxP3 (1:100, 236A/E7, Thermo Fisher) and anti-CTLA4 (1:100, BNI3, Abcam). Expression of PD-L1 and PD-L2 was investigated on tumor and immune cells. CD8, CD4, CD56, FoxP3, CTLA-4, and PD1 staining were used to further characterize the lymphocytes.

### Scoring of Tumor Infiltrating Lymphocytes

The Immunoscore was investigated according to criteria formulated by Clark et al (19). In brief, lymphocytes were classified as brisk if they diffusely infiltrated the entire invasive component and were interposed between melanoma cells or if they were present alongside the entire base of tumor. Lymphocytes were classified as nonbrisk if they focally infiltrated the tumor and were not present along the entire tumor base. If no lymphocytes were present or if lymphocytes did not infiltrate the tumor, they were classified as absent.

Lymphocytes were morphologically identified by H&E while subtyping of the lymphocytic infiltrate was performed by staining for CD8, CD4, CD56, FoxP, CTLA-4, PD1, PD-L1, and PD-L2.

### Quantification of Lymphocytic Subtypes

Percentage of lymphocytes positive for CD8, CD4, CD56, Fox P3, CTLA-4, and PD-1 was calculated according to the total number of tumor infiltrating lymphocytes in a sample. Additionally, we determined ratios of CD4- and CD8-positive lymphocytes. Geographical associations of lymphocytic subtypes and tumor cells could not be investigated due to TMA used for the majority of samples. PD-L2 was evaluated as described below for PD-L1 immunohistochemistry.

### Quantification of PD-L1 Expression

In order to determine the most specific PD-L1 expression pattern, we evaluated IHC obtained using four well-established anti-PD-L1 clones (E1L3N, cell signaling; SP263, Roche; SP142, Roche; 28.8, Abcam). Thereafter, PD-L1 staining in tumor cells was considered positive if staining was membranous, regardless of intensity. Tumors were defined as positive if they contained ≥5% PD-L1 positive tumor cells. Expression of PD-L1 in tumor infiltrating lymphocytes was evaluated by measuring the area of PD-L1 positive lymphocytes from the whole tumor area (20).

### Statistical Analysis

Fisher's exact test was used to assess the differences in the distribution of brisk vs. nonbrisk lymphocytes between primary melanomas and metastases (including those present prior to initiation of treatment and those which developed during treatment.

Kaplan-Meier curves were used to determine overall survival and progression-free survival depending on the Immunoscore, PD-L1/PD-L2 expression of tumor and immune cells, the different lymphocytic subtypes and CD4/CD8-ratio. Data were statistically proved by log-rank tests.

*T*-tests were used to compare the mean expression between patients with or without progression during anti-PD1-therapy. Statistical tests were performed within the same tumor groups (primary tumors, lymph node metastases and distant metastases before and after checkpoint-inhibitor therapy).

All statistical analyses were performed using SPSS 2.0. *p* levels < 0.05 were considered significant.

## Results

### Immune Infiltration Is Significantly Increased in Primary Melanoma When Compared to That Seen in Metastases

**Figure 1:**
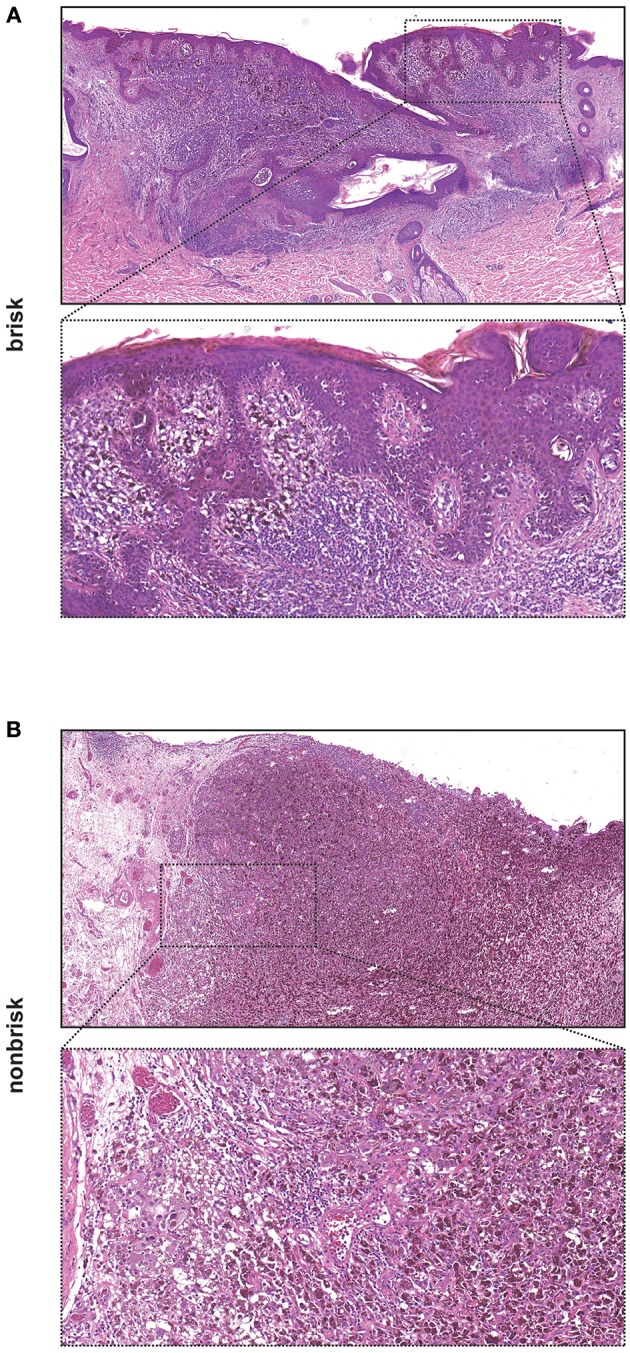
Images of a melanoma with brisk and nonbrisk lymphocytic infiltration. **(A)** Brisk melanoma with entire base of the tumor surrounded by a dense band-like lymphocytic infiltration [H&E, original magnification x40 and x130 (insert)]. **(B)** Nonbrisk melanoma with only focal lymphocytic infiltration [H&E, original magnification x40 and x130 (insert)].

**Figure 2:**
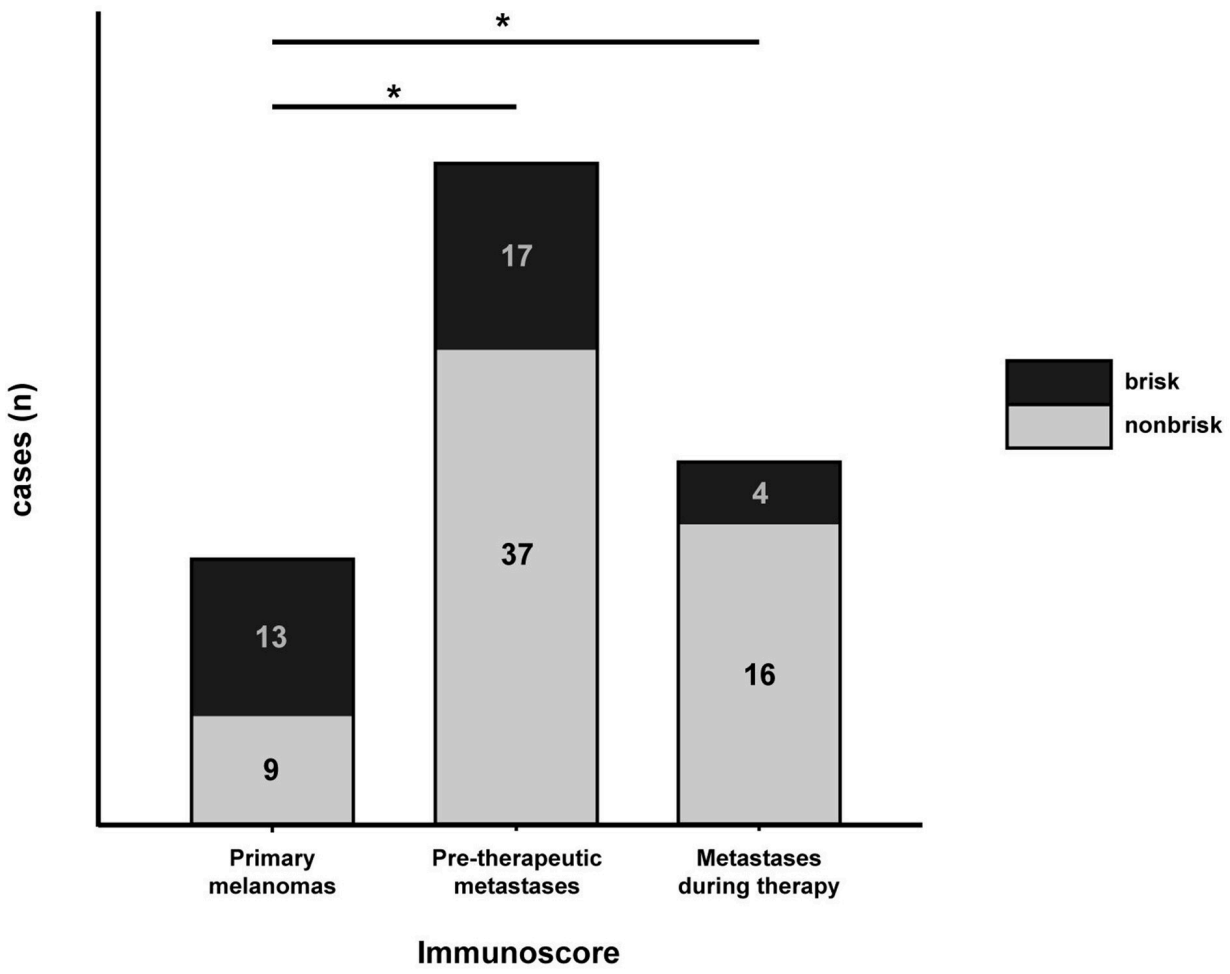
Distribution of brisk vs. nonbrisk infiltration in primary melanomas, pre-therapeutic metastases and metastases which developed during anti-PD-1-therapy. Number of brisk cases is indicated in black and of nonbrisk cases in gray fields. Statistical significance between investigated groups was determined by Fischer's exact test (^*^p < 0.05).

We first determined the Immunoscore based on lymphocytic infiltration, classifying the tumors into absent, brisk and nonbrisk groups (Figure 1). We assessed the Immunoscore in a total of 22 samples of primary melanomas; 13 (59.1%) were classified as brisk and 9 (40.9%) samples were classified as nonbrisk. We additionally analyzed 88 metastases out of which 54 were obtained before treatment and 20 were obtained post treatment with anti-PD-1-therapy. Seventeen (31.5%) pre-therapeutic metastases (metastases present before any treatment) were classified as brisk and 37 (68.5%) as nonbrisk. In the cohort of metastases which developed during treatment, 4 (20%) were classified as brisk while 16 (80%) were classified as nonbrisk (Figure 2). The remaining metastases (n = 14) that were obtained after initial Ipilimumab therapy in patients that had not undergone Nivolumab/Pembrolizumab therapy were not included in the Immunoscoring.

In order to investigate the differential distribution of lymphocytic infiltration we compared brisk status in primary melanomas (prior to anti-tumor therapy) vs. pre-therapeutic metastases as well as between primary melanomas and metastases which developed during anti-PD-1-therapy. Immune infiltration was not only significantly increased in primary melanomas when compared to pre-therapeutic metastases (*p* = 0.0381), but also increased when immune infiltration in the primary melanomas was compared to that in metastases developed during treatment (*p* = 0.0135; Figure 2).

**Figure 3:**
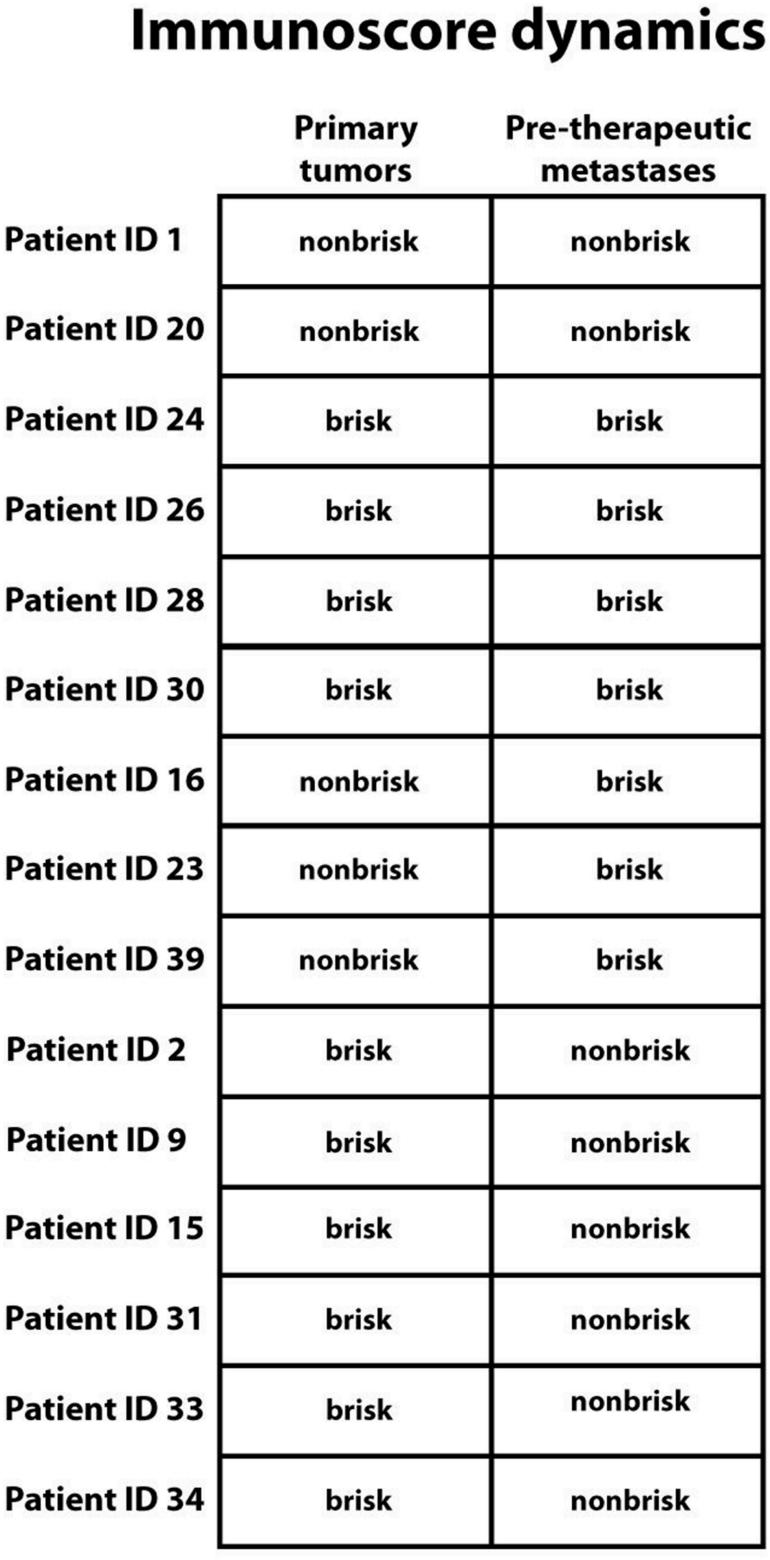
Immunoscore dynamics between primary melanomas and pre-therapeutic metastases in the same patient. Top down: No change in Immunoscore was seen in 6 patients, increased Immunoscore in metastases was seen in 3 patients, and a decreased Immunoscore in metastases was seen in 6 patients. Patient identification numbers are denoted besides.

Next, we compared the Immunoscore in primary melanomas to that in pre-therapeutic metastases in the same patient (intra-individual immune cell infiltration). In 40% of cases there was no difference in the Immunoscore (6/15 patients). Whilst there was an increased metastatic Immunoscore in 20% of cases (3/15), in the remaining 40% (6/15) there was increased Immunoscore in the primary melanoma when compared to that in the pre-therapeutic metastases (Figure 3 and Tables 2, 3). Due to low number of metastases which developed during checkpoint therapy, we were not able compare Immunoscores from pre-therapeutic metastases to the Immunscore in metastases which developed during checkpoint therapy in the same patient.

**Table 2:** Immunscore and PD-L1 expression before and after anti-PD1 therapy.

**Patient ID**	**Pre-therapeutic tissue**	**n**	**PD-L1 expression (mean%)**	**Immunoscore**	**Metastases during therapy**	**n**	**PD-L1 expression (mean %)**	**Immunoscore**
6	primary tumor	1	5	2	distant metastases	1	0	2
8	primary tumor	1	20	2	lymphe node metastases	3	60	2
9	distant metastases	1	<1	1	distant metastases	6	<1	1
11	satellite metastases	2	2	2	distant metastases	1	10	1
13	not available	X	X	X	distant metastases	1	15	1
14	distant metastases	1	20	1	distant metastases	1	30	1
21	lymphe node metastases	1	25	2	lymphe node- and distant metastases	7	0	1

**Table 3:** Immunscore and PD-L1 expression before and after anti CTLA-4 therapy.

**Patient ID**	**Pre-therapeutic tissue**	**n**	**PD-L1 expression (mean%)**	**Immunoscore**	**Metastases during therapy**	**n**	**PD-L1 expression (mean %)**	**Immunoscore**
2	lymph node- and distant metastases	3	15	1	distant metastases	2	35	1
3	primary tumor	1	0	1	distant metastases	1	<1	1
4	lymphe node metastases	2	30	1	distant metastases	1	0	1
9	distant metastases	1	<1	1	distant metastases	1	2	1
12	not available	X	X	X	distant metastases	8	<1	1
21	lymphe node metastases	1	25	2	distant metastases	1	15	1

### The Immunoscore Is Associated With Improved Overall Survival During Checkpoint Therapy

**Figure 4:**
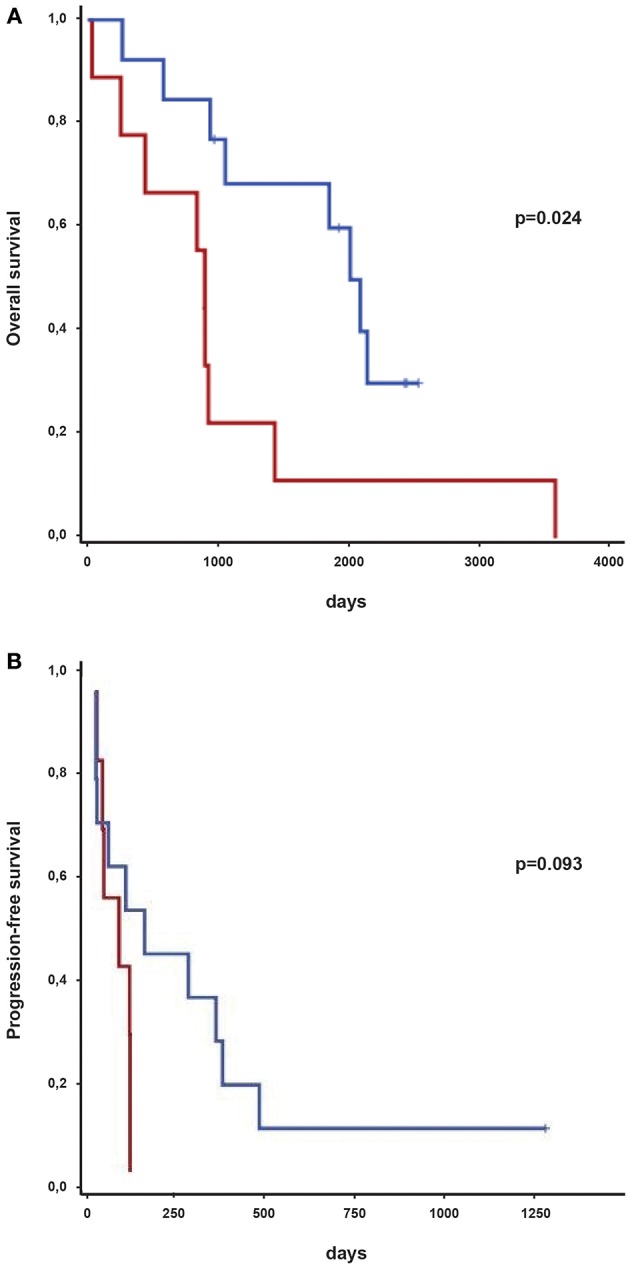
Survival of melanoma patients treated with immune-checkpoint inhibitors depending on immune infiltration of primary tumors classified as brisk or nonbrisk. **(A)** Kaplan-Meier curves indicating overall survival of brisk (in blue) and nonbrisk (in red) primary tumors from melanoma patients. Survival groups were compared by log-rank test. *p*-values are indicated. **(B)** Kaplan-Meier curves indicating progression-free survival of brisk (in blue) and nonbrisk (in red) primary tumors from melanoma patients. Survival groups were compared by log-rank test. p-values are indicated.

Next, we aimed to determine whether the Immunoscore was associated with overall survival in melanoma. This was chosen as the most clinically significant parameter. We observed a statistically significant increase in overall survival in patients with a brisk lymphocytic infiltrate compared to patients with a nonbrisk infiltrate of their primary tumors (*p* = 0.024; Figure 4A). 5 year-survival rate for patients with a brisk tumor infiltrate and a nonbrisk infiltrate was 59.8 and 11.1%, respectively. Concordantly, we observed a trend in increased progression-free survival progression free survival of patients with a brisk lymphocytic tumor-infiltrate compared to patients with a nonbrisk infiltrate (*p* = 0.093; Figure 4B).

An association between the Immunoscore and survival rates could be demonstrated when evaluating primary melanomas, but there was no association between the Immunoscore in metastases and survival. Moreover, subtyping lymphocytic infiltrate using CD8, CD4, CD56, FoxP3, CTLA-4, PD-1, PD-L1, or PD-L2 expression did not lead to significant associations with overall survival (data not shown).

### The Impact of BRAF Mutation Status on Clinical Outcome

**Figure 5:**
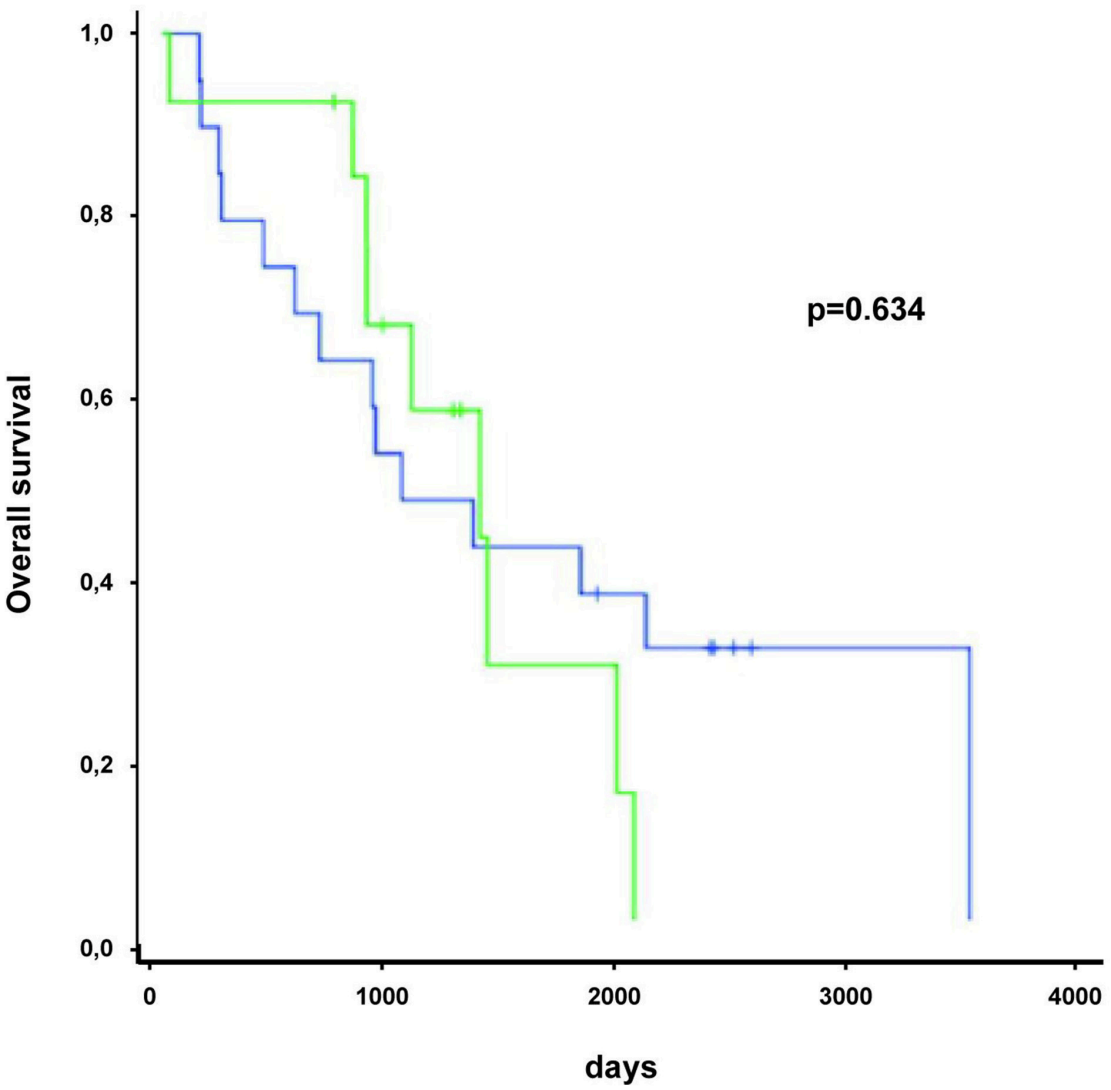
BRAF mutation status in patients undergoing immune checkpoint therapy was not significantly associated with overall survival. Green line represents patients with BRAF mutation, blue line indicates patients without BRAF mutation.

We further evaluated the association of BRAF mutations with clinical outcome. BRAF mutation status was investigated in context of diagnostic work-up and not specifically for the current study. 20 (62.5%) out of 32 patients showed wt BRAF and 12 (37.5%) harbored mutations in the BRAF gene. Out of these 12 cases, 10 had the V600E mutation, 1 exhibited the D594V mutation and a further patient had the L597Q mutation. There was no association between BRAF status and either overall survival or progression-free in our melanoma cohort. We also observed no association between BRAF status and Immunoscore and/or PD-L1 status (Table 4 and Figure 5).

**Table 4:** Association between BRAF status und immunoscore.

		**Immunoscore**		
**BRAF-status**		**Nonbrisk**	**Brisk**	**Total**
Wildtype	n	4	10	14
Mutation	n	5	3	8
Total	n	9	13	22
				*p* = 0.187

### The PD-L1 Antibody Clone SP263 Demonstrated the Highest Immunohistochemical Specificity

**Figure 6:**
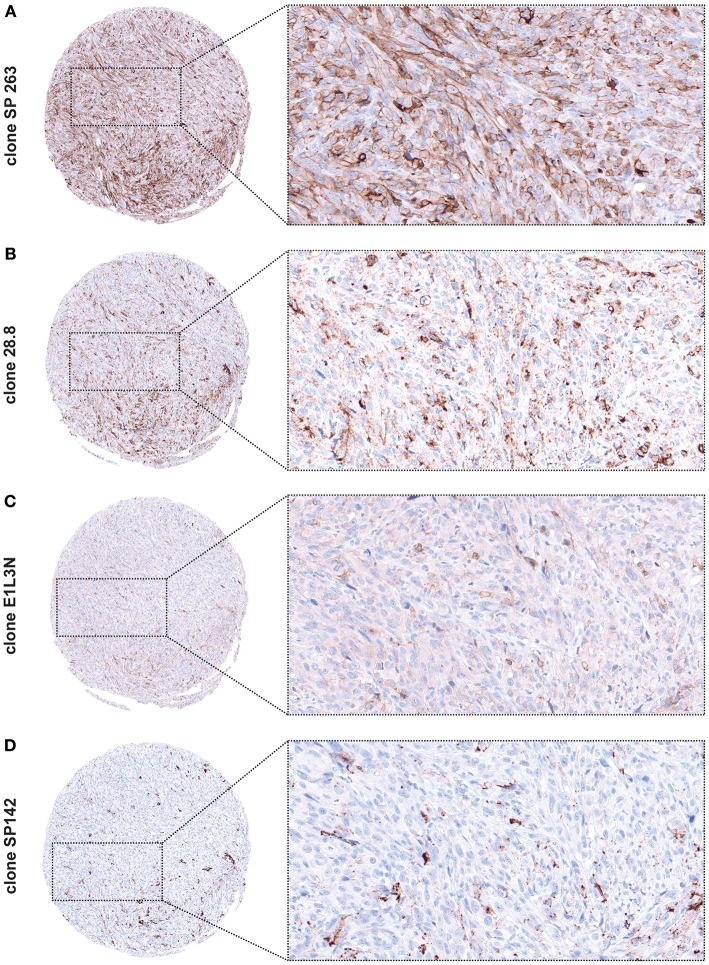
PD-L1 expression using different anti-PD-L1 clones demonstrated on the same tumor core [original magnification x84 and x300 (insert)]. **(A)** Clone SP263 stains the highest proportion of tumor cells and shows the strongest expression. **(B)** Clone 28.8. shows weaker expression and additionally a granular background. **(C)** Clone E1L3N shows weak expression with a discreet staining of cell membranes. **(D)** Clone SP142 shows the weakest expression (black pigment accord to melanin pigment).

In order to determine the optimal protocol for determining PD-L1 expression in melanoma, we tested four distinct anti-PD-L1 clones, namely SP263, 28.8, E1L3N, and SP142 in 22 primary melanomas and 88 metastases. Two metastases were excluded from the results due to exhaustion of tissue material during the immunohistochemical staining. We observed strikingly different staining patterns as representatively shown in Figure 6. The percentage of PD-L1 positive tumor cells in the same investigated sample varied from 100% (clone SP263) to 0% (clone SP142). Using clone 28.8 and E1L3N, 60 and 20% respectively of tumor cells were PD-L1 positive. When comparing PD-L1 expression in primary tumors vs. metastases using the four antibody clones the results were also divergent. Specifically, in 22 cases of primary tumors, half (n = 11) were interpreted as PD-L1 positive by using clone SP263 (Table 5). On the other hand, by using clones 28.8, E1L3N and SP142, we observed 3 (13.6%), 3 (13.6%), and 1 (4.5%) positive cases, respectively. Mean PD-L1 expression value of positive tumor cells for clone SP263 was 11.5%, for clone 28.8 was 3.63%, for clone E1L3N was 3.75% and for SP142 was 2.27%. Expression range reached from 0 to 100 positive tumor cells for clone SP263, from 0 to 40 for clone 28.8, from 0 to 70 for clone E1L3N und 0-50 for clone SP142. When investigating metastases for PD-L1 expression, we observed a similar pattern. By using clone SP263, we observed 27 (31.4%) positive cases while for clones 28.8, E1L3N and SP142, we observed 11 (12.8%), 4 (4.7%), and 4 (4.7%), respectively. Mean PD-L1 expression value of positive tumor cells for clone SP263 was 12.9%, for clone 28.8 was 5.3%, for clone E1L3N was 1.7% and for SP142 was 1.1%. Expression range reached from 0 to 100 positive tumor cells for clone SP263, from 0 to 95 for clone 28.8, from 0-80 for clone E1L3N und 0-40 for clone SP142.

**Table 5:** PD-L1 expression using different anti-PD-L1 clones.

	**Mean PD-L1 expression**	**Range**	**Number of positive cases**	**Number of negative cases**	**Positive cases (%)**	**Negative cases (%)**
**Primary tumors (*****n*** **=** **22)**						
SP263	11.5	0–100	11	11	50	50
Abcam 28.8	3.63	0–40	3	19	13.6	86.4
E1L3N	3.75	0–70	3	19	13.6	86.4
SP142	2.27	0–50	1	21	4.5	95.5
**Metastases (*****n*** **=** **86)**						
SP263	12.9	0–100	27	59	31.4	68.6
Abcam 28.8	5.3	0–95	11	75	12.8	87.2
E1L3N	1.7	0–80	4	82	4.7	95.3
SP142	1.1	0–40	4	82	4.7	95.3

Overall, clone SP263 showed highest specificity and the strongest staining intensity of PD-L1 in both primary tumors and metastases. Conversely, clones 28.8 and E1L3N showed weaker staining intensity with a discrete staining of the cell membranes while clone 28.8 showed additional granular background staining. Clone SP142 showed the weakest staining intensity as well as the lowest frequency of positive tumor cells. These observations supported the use of clone SP263 for further investigations.

### PD-L1 Expression Is Not Associated With Overall Survival

**Figure 7:**
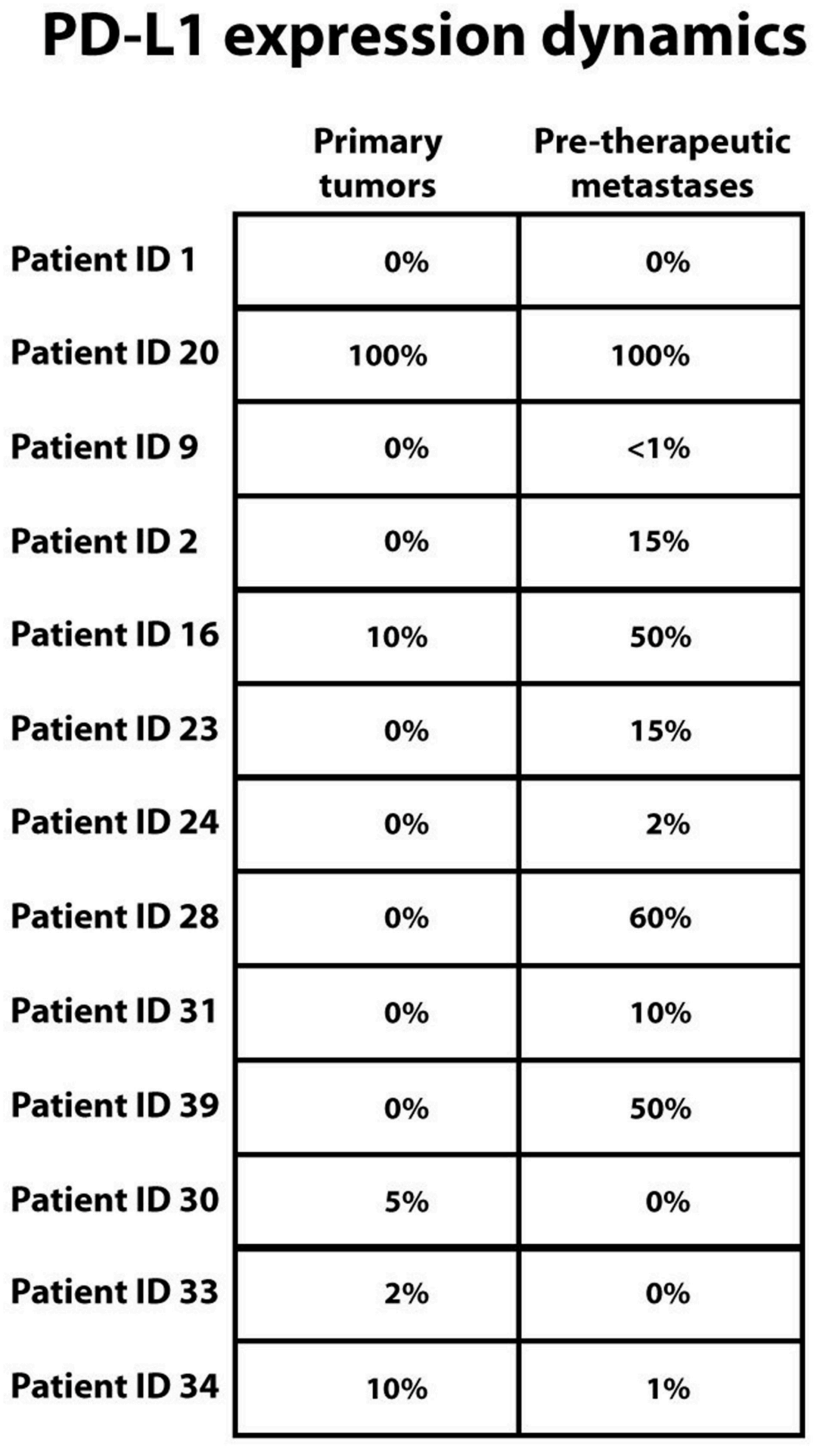
PD-L1 melanoma expression dynamics between primary melanomas and pre-therapeutic metastases in the same patient. Top down: No change in PD-L1 expression was seen in 3 patients, higher PD-L1 expression in metastases was seen in 7 patients and lower PD-L1 expression in metastases was seen in 3 patients. PD-L1 expression (clone SP 263) is reported as percentage of PD-L1 positive tumor cells from all tumor cells. In case of more than one metastasis, mean value is stated. Patient identification numbers are denoted besides. For patients with identification number 15 and 26 evaluation of PD-L1 expression was not possible.

Given that the immune infiltration was significantly higher in primary melanomas when compared to pre-therapeutic metastases, we next sought to determine intra-individual PD-L1 expression in primary melanomas and untreated metastases. We were able to evaluate PD-L1 expression in primary melanomas and untreated metastases in 13 out of 15 patients. There was no difference in PD-L1 expression in 3 (23.1%) cases, PD-L1 was upregulated in metastases in 7 (53.8%) cases, and higher PD-L1 expression in primary tumors, when compared to metastases obtained before immunotherapy, was present in 3 cases (23.1%) (Figure 7).

There was no correlation between PD-L1 expression and overall survival of melanoma patients treated with immune checkpoint inhibitors.

We also investigated any possible association between PD-L1 expression and the Immunoscore but observed no statistically significant difference in PD-L1 status between the brisk and nonbrisk groups.

## Discussion

The treatment of metastatic melanoma continues to represent a major clinical challenge, not only due to the aggressive nature of the disease, but also due to the potentially life-threatening side-effects associated with immunotherapy. However, the development of immune checkpoint inhibitors has markedly increased our therapeutic armamentarium and translated into impressive improvements in overall survival. Unfortunately, a significant proportion of patients still fail to respond to treatment. In order to determine which patients may respond best to checkpoint inhibition we retrospectively analyzed immune cell infiltration and PD-L1 status in a cohort of melanoma treated with CTLA-4 and/or anti-PD-1 inhibitors. Increased tumor immune infiltration in primary melanomas (measured by the Immunoscore) prior to immune checkpoint inhibition was associated with improved overall patient survival (Figure 4). Interestingly, increased recruitment of cytotoxic CD8+ lymphocytes alone was not observed in the favorable “brisk” setting (data not shown). Furthermore, no significant difference was observed in the number of CD4+ helper cells between the brisk and nonbrisk groups and consequently no difference was observed in CD4+/CD8+ ratio between two settings (data not shown).

However, the long-term benefit of immune checkpoint inhibition was evidenced by increased overall survival in patients that harbored highly infiltrated primary melanomas. It is important to bear in mind that response, overall survival and progression-free survival are independent parameters. For example, response may vary over time, especially in the context of acquired resistance and progression-free survival does not necessarily equate with improved overall survival (see Supplementary Table 1). Furthermore, there is an important and recognized difference between predictors of treatment response and prognostic markers (8). In this context, the Immunoscore represents a novel prognostic marker of treatment response, but is not a suitable stand-alone parameter to predict which patients will clinically benefit from checkpoint inhibition.

In contrast to previous studies (1, 17), we did not find an association between CD8+ cell infiltration with response to checkpoint inhibition in melanoma patients. In fact, Madonna et al. (21) reported low densities of CD8+ lymphocytes at the tumor periphery and an association with response to Ipilimumab. PD-L1 status was not a predictive marker for survival or treatment response.

Whilst the reason for the divergent results in terms of CD8+ infiltration is unclear, it is important to draw attention to the methodological differences between the studies. For example, Tumeh et al. (13) investigated patients who underwent PD-1 (Pembrolizumab) monotherapy, albeit in three difference dosing schedules, and Madonna et al examined patients treated with Ipilimumab. Our “real world” cohort was more heterogeneous in terms of treatment modality (PD-1/CTLA4 monotherapy vs. PD-1/CTLA4 combined therapy) which may have influenced CD8+ cell infiltration. Large, prospective studies would be required to determine the extent to which CD8+ cell infiltration in treatment type dependent. Moreover, it would be interesting to determine the extent to which PD-L1 expression on peripheral T cells correlates with intratumoural T cell PD-L1 expression, given the association between circulating T cell PD-L1 expression and response to checkpoint inhibition (22, 23). We cannot exclude that the Immunoscore reflects a pre-existing anti-melanoma T cell response. However, this would be difficult to experimentally and/or clinically confirm or refute given that we do not have a patient cohort remain untreated. In any case, given that the Immunoscore in metastases was *not* associated with improved overall survival in our study, such pre-existing anti-melanoma T cell responses would have been limited to the primary tumor.

Next, we investigated BRAF mutation status in the context of absent, brisk and non-brisk Immunoscores in primary tumors and overall survival. BRAF status was not predictive for overall survival of melanoma patients during immune checkpoint therapy. The frequency of BRAF mutation in our cohort was similar to previous melanoma cohorts (24). Whilst there is conflicting data regarding the effect of BRAF status on treatment outcome during checkpoint immunotherapy, our study is in line with studies showing that Nivolumab treatment efficacy irrespective of BRAF status (25). It is currently unclear whether initial therapy with BRAF/MEK inhibitors followed by immune checkpoint therapy, or vice versa, translates to improved overall rates of survival for patients with the BRAF mutation.

Nevertheless, our study highlights the utility of the Immunoscore, a robust and readily available scoring tool, which is associated with overall survival in patients with metastastic melanoma undergoing immune checkpoint therapy.

We also aimed to clarify whether the pattern of tumor immune cell infiltration differs between primary melanoma, untreated metastases and metastases which developed during treatment with immune checkpoint inhibition. Due to more aggressive nature of metastastic melanoma, we expected to observe an increased immune-cell infiltration in primary melanomas when compared to that in metastases. Indeed, the pattern of immune-cell infiltration was dramatically different between primary melanomas and both metastases subgroups, in line with our hypothesis (Figure 2). We then sought to compare the Immunoscore from primary tumors and metastases in individual patients. Although we observed generally a lower Immunoscore in untreated metastases, there was no reduction in tumor immune cell infiltration when comparing the primary melanoma to the metastases in every patient, probably due to the heterogenous nature of these tumors (Figure 3).

Theoretically, both immune cell tumor infiltration and expression of PD-L1 on tumor cells are required for successful anti-PD-1 and anti-PD-L1 checkpoint therapy. Therefore, we further investigated the correlation of tumor PD-L1 status in the context of the brisk and nonbrisk group as well as alone on the survival of melanoma patients after anti-PD-1 immunotherapy. We found no significant correlations in either setting (9). We also observed no correlation of PD-L1 expression dynamics in matching primary melanomas and corresponding metastases of the same patient. Again, it is important to note (i) the variations in immune-checkpoint inhibitor treatment regiments (single vs. combined anti-CTLA-4 and anti-PD-1/PD-L1 vs. sequential combined anti-CTLA-4 and anti-PD-1/PD-L1 immuno-checkpoint inhibition), (ii) the various antibodies used to detect tumor PD-L1 status, (iii) the tumor type (predominantly cutaneous melanoma as opposed to mucosal and/or acral) and (iv) the small size of metastases when taking our data into account. We could demonstrate that PD-L1 expression was heavily dependent on the PD-L1 antibody clone which was used, perhaps partially explaining the, at times, confounding effect of PD-L1 expression reported in the literature (Figure 6 and Table 5). Based on our data, we selected and employed the most specific clone (263) and the overall level of melanoma PD-L1 expression in our study was similar to that reported in the literature (26, 27).

In conclusion, the results of our study suggest that total tumor immune infiltration, not PD-L1 status, is important for predicting the survival of melanoma patients undergoing checkpoint inhibitor therapy. However, this may be specific to our cohort where many melanoma patients were pretreated with Ipilimumab prior to administering Nivolumab/Pembrolizumab (see Supplementary Table 1). Whilst our results require replication in a large, prospective study, they provide evidence that the Immunoscore, a validated and easy to use tool, which does not require laborious and potentially erroneous cell counting, is a novel marker for survival in melanoma patients treated with immune checkpoint therapy. Provided that our findings can be replicated in larger, prospective studies, the Immunscore may represent an inexpensive, simple and robust tool which can be rapidly incorporated into routine clinico-pathological practice.

## Author Contributions

PT, SP, and CK: planned the research project. CK, MJ, AO, and WV: performed the pathological staining and data analysis. CK, MJ, EL, OH, VG, SP, and PT: wrote and/or revised the manuscript.

### Conflict of Interest Statement

PT has received speaker's honoraria from BMS, Novartis, and Roche, consultant's honoraria from BMS, Merck, Novartis, and Roche and travel support from BMS, and Roche. EL has received speakers' honoraria from Novartis. The remaining authors declare that the research was conducted in the absence of any commercial or financial relationships that could be construed as a potential conflict of interest.

## Supplementary Material

Supplementary Table 1 Immunoscore, treatment history and best response rate in the cohort.

## References

[1] Ribas A Wolchok JD. Cancer immunotherapy using checkpoint blockade. Science. (2018) 359:1350–5. 10.1126/science.aar4060 29567705 PMC7391259

[2] Gide TN Wilmott JS Scolyer RA Long GV. Primary and acquired resistance to immune checkpoint inhibitors in metastatic melanoma. Clin Cancer Res. (2018) 24:1260–70. 10.1158/1078-0432.CCR-17-2267 29127120

[3] Tarhini A Kudchadkar RR. Predictive and on-treatment monitoring biomarkers in advanced melanoma:Moving toward personalized medicine. Cancer Treat Rev. (2018) 71:8–18. 10.1016/j.ctrv.2018.09.005 30273812

[4] Blank CU Haanen JB Ribas A Schumacher TN. CANCER IMMUNOLOGY. The “cancer immunogram.” Science. (2016) 352:658–60. 10.1126/science.aaf2834 27151852

[5] Schumacher TN Schreiber RD. Neoantigens in cancer immunotherapy. Science. (2015) 348:69–74. 10.1126/science.aaa4971 25838375

[6] Hugo W Zaretsky JM Sun L Song C Moreno BH Hu-Lieskovan S. Genomic and transcriptomic features of response to anti-PD-1 therapy in metastatic melanoma. Cell. (2016) 165:35–44. 10.1016/j.cell.2016.02.065 26997480 PMC4808437

[7] Carbognin L Pilotto S Milella M Vaccaro V Brunelli M Caliò A. Differential activity of nivolumab, pembrolizumab and MPDL3280A according to the tumor expression of programmed Death-Ligand-1 (PD-L1): sensitivity analysis of trials in melanoma, lung and genitourinary cancers. PLoS ONE. (2015) 10:e0130142. 10.1371/journal.pone.0130142 26086854 PMC4472786

[8] Jessurun CAC Vos JAM Limpens J Luiten RM. Biomarkers for response of melanoma patients to immune checkpoint inhibitors: a systematic review. Front Oncol. (2017) 7:233. 10.3389/fonc.2017.00233 29034210 PMC5625582

[9] Wolchok JD Chiarion-Sileni V Gonzalez R Rutkowski P Grob JJ Cowey CL. Overall survival with combined nivolumab and ipilimumab in advanced melanoma. N Engl J Med. (2017) 377:1345–56. 10.1056/NEJMoa1709684 28889792 PMC5706778

[10] Buder-Bakhaya K Hassel JC. Biomarkers for clinical benefit of immune checkpoint inhibitor treatment-a review from the melanoma perspective and beyond. Front Immunol. (2018) 9:1474. 10.3389/fimmu.2018.01474 30002656 PMC6031714

[11] Robert C Long GV Brady B Dutriaux C Maio M Mortier L. Nivolumab in previously untreated melanoma without BRAF mutation. N Engl J Med. (2015) 372:320–30. 10.1056/NEJMoa1412082 25399552

[12] Aguiar PN Jr Santoro IL Tadokoro H de Lima Lopes G Filardi BA Oliveira P. The role of PD-L1 expression as a predictive biomarker in advanced non-small-cell lung cancer:a network meta-analysis. Immunotherapy. (2016) 8:479–88. 10.2217/imt-2015-0002 26973128

[13] Tumeh PC Harview CL Yearley JH Shintaku IP Taylor EJ Robert L. PD-1 blockade induces responses by inhibiting adaptive immune resistance. Nature. (2014) 515:568–71. 10.1038/nature13954 25428505 PMC4246418

[14] Azimi F Scolyer RA Rumcheva P Moncrieff M Murali R McCarthy SW. Tumor-infiltrating lymphocyte grade is an independent predictor of sentinel lymph node status and survival in patients with cutaneous melanoma. J Clin Oncol. (2012) 30:2678–83. 10.1200/JCO.2011.37.8539 22711850

[15] Thomas NE Busam KJ From L Kricker A Armstrong BK Anton-Culver H. Tumor-infiltrating lymphocyte grade in primary melanomas is independently associated with melanoma-specific survival in the population-based genes, environment and melanoma study. J Clin Oncol. (2013) 31:4252–9. 10.1200/JCO.2013.51.3002 24127443 PMC3821014

[16] Kluger HM Zito CR Barr ML Baine MK Chiang VL Sznol M. Characterization of PD-L1 expression and associated t-cell infiltrates in metastatic melanoma samples from variable anatomic sites. Clin Cancer Res. (2015) 21:3052–60. 10.1158/1078-0432.CCR-14-3073 25788491 PMC4490112

[17] Madore J Vilain RE Menzies AM Kakavand H Wilmott JS Hyman J. PD-L1 expression in melanoma shows marked heterogeneity within and between patients:implications for anti-PD-1/PD-L1 clinical trials. Pigment Cell Melanoma Res. (2015) 28:245–53. 10.1111/pcmr.12340 25477049

[18] Daud AI Wolchok JD Robert C Hwu WJ Weber JS Ribas A. Programmed death-ligand 1 expression and response to the anti-programmed death 1 antibody pembrolizumab in melanoma. J Clin Oncol. (2016) 34:4102–9. 10.1200/JCO.2016.67.2477 27863197 PMC5562434

[19] Clark WH Jr Elder DE Guerry D IV Braitman LE Trock BJ Schultz D. Model predicting survival in stage I melanoma based on tumor progression. J Natl Cancer Inst. (1989) 81:1893–904. 10.1093/jnci/81.24.1893 2593166

[20] Scheel AH Dietel M Heukamp LC Jöhrens K Kirchner T Reu S. [Predictive PD-L1 immunohistochemistry for non-small cell lung cancer:Current state of the art and experiences of the first German harmonization study]. Pathologe. (2016) 37:557–67. 10.1007/s00292-016-0189-1 27510417

[21] Madonna G Ballesteros-Merino C Feng Z Bifulco C Capone M Giannarelli D. PD-L1 expression with immune-infiltrate evaluation and outcome prediction in melanoma patients treated with ipilimumab. Oncoimmunology. (2018) 7:e1405206. 10.1080/2162402X.2017.1405206 30524879 PMC6279420

[22] Takeuchi Y Tanemura A Tada Y Katayama I Kumanogoh A Nishikawa H. Clinical response to PD-1 blockade correlates with a sub-fraction of peripheral central memory CD4+ T cells in patients with malignant melanoma. Int Immunol. (2018) 30:13–22. 10.1093/intimm/dxx073 29294043

[23] Jacquelot N Roberti MP Enot DP Rusakiewicz S Ternès N Jegou S. Predictors of responses to immune checkpoint blockade in advanced melanoma. Nat Commun. (2017) 8:592. 10.1038/s41467-017-00608-2 28928380 PMC5605517

[24] Robert C Schachter J Long GV Arance A Grob JJ Mortier L. Pembrolizumab versus Ipilimumab in advanced melanoma. N Engl J Med. (2015) 372:2521–32. 10.1056/NEJMoa1503093 25891173

[25] Larkin J Lao CD Urba WJ McDermott DF Horak C Jiang J. Efficacy and safety of nivolumab in patients with BRAF V600 mutant and BRAF wild-type advanced melanoma:a pooled analysis of 4 clinical trials. JAMA Oncol. (2015) 1:433–40. 10.1001/jamaoncol.2015.1184 26181250

[26] Wolchok JD Kluger H Callahan MK Postow MA Rizvi NA Lesokhin AM. Nivolumab plus ipilimumab in advanced melanoma. N Engl J Med. (2013) 369:122–33. 10.1056/NEJMoa1302369 23724867 PMC5698004

[27] Morrison C Pabla S Conroy JM Nesline MK Glenn ST Dressman D. Predicting response to checkpoint inhibitors in melanoma beyond PD-L1 and mutational burden. J Immunother Cancer. (2018) 6:32. 10.1186/s40425-018-0344-8 29743104 PMC5944039

